# A Novel Model to Simulate Flexural Complements in Compliant Sensor Systems

**DOI:** 10.3390/s18041029

**Published:** 2018-03-29

**Authors:** Hongyan Tang, Dan Zhang, Sheng Guo, Haibo Qu

**Affiliations:** 1 School of Mechanical, Electronic and Control Engineering, Beijing Jiaotong University, Beijing 100044, China; 14116352@bjtu.edu.cn (H.T.); shguo@bjtu.edu.cn (S.G.); hbqu@bjtu.edu.cn (H.Q.); 2 Lassonde School of Engineering, York University, Toronto, ON M3J 1P3, Canada

**Keywords:** dynamic spline pseudo-rigid-body model, flexural beam, force sensor, compliant mechanisms, large deflection

## Abstract

The main challenge in analyzing compliant sensor systems is how to calculate the large deformation of flexural complements. Our study proposes a new model that is called the spline pseudo-rigid-body model (spline PRBM). It combines dynamic spline and the pseudo-rigid-body model (PRBM) to simulate the flexural complements. The axial deformations of flexural complements are modeled by using dynamic spline. This makes it possible to consider the nonlinear compliance of the system using four control points. Three rigid rods connected by two revolute (R) pins with two torsion springs replace the three lines connecting the four control points. The kinematic behavior of the system is described using Lagrange equations. Both the optimization and the numerical fitting methods are used for resolving the characteristic parameters of the new model. An example is given of a compliant mechanism to modify the accuracy of the model. The spline PRBM is important in expanding the applications of the PRBM to the design and simulation of flexural force sensors.

## 1. Introduction

A flexural force sensor is a mechanism that uses the deformation of its flexible components to gain force signals. For rigid body mechanisms, there are some inevitable expectation, such as high precision, speed, efficiency, and performance, which represent great challenges for sensor systems design. Frictions and clearances are two main factors influencing the accuracy and dynamic performances of moment and force sensors. In addition, the assembly requires an enormous portion of the product expenses. These problems are not easy to solve for traditional rigid body mechanisms. Nevertheless, compliant mechanisms possess many advantages for expense reduction and performance improvement of sensor function, such as fabrication processes, wear, friction, and noise reduction. All of these characteristics make compliant mechanisms promising candidates to be used in force sensor systems [1,2,3].

The main challenge in analyzing compliant mechanisms is how to simulate the large deflection of flexural components. There have been several efforts [4,5,6] to solve such simulation problems [7], e.g., the pseudo-rigid-body method [8], the elliptic integral solution [9,10], the circle-arc method [11], the domain decomposition method [12,13], and the chain algorithm [14]. Borboni et al. [4] discussed the large deflection of a cantilever beam made of a non-linear material. However, the research of martials’ deflection in robotic, mechanism, and sensor field focused on linear deflection analysis for industrial applications. In Reference [15], a method with linear torsion springs was applied to simulate the flexible component. The result of this study became the fundamental principle of the pseudo-rigid-body model (PRBM). Howell and Midha [14,16] proposed the PRBM, thus simplifying the flexible component. The PRBM is based on the analysis of the rigid body mechanism. The 1 Revolute (1R) PRBM, which is forced by three different end loads, was calculated in Reference [17]. Su [18] proposed a 3R PRBM, which improved the simulation of the flexural component with combined loads. The 3R PRBM possesses higher simulation precision, but it is still difficult to find the inverse kinematic and the characteristic parameters of the model.

As the 1R and 3R PRBM contain pin joints, they can thus function as the bending deformation. When the combined loads are subjected to a flexible beam, it is impossible to calculate the axial deformation by PRBMs with revolute pairs only. In Reference [19], the lateral and axial deformation of a flexible link were simulated by a Prismatic-Revolute (PR) PRBM with a revolute pair and a prismatic pair. In Reference [20], a Prismatic-Revolute-Revolute (PRR) PRBM with a sliding link and two rotating links was proposed to simulate beam in large deformation with combined moment loads and end force. The PRBMs achieved a good balance between accuracy and simplicity. However, it presented two main drawbacks. The first drawback is that the simulation results in dynamic may be very inaccurate because these models are designed for static and quasi-static assessment. The second is that it is too difficult to model a three-dimensional (3D) structure with complex shapes by using simple rigid bodies.

A beam can be considered as a dynamic system with elasticity and mass for which the number of degrees-of-freedom (DOF) is infinite. The number of DOF can be reduced by a few variables in the modeling strategies. Shabana [21,22] proposed a flexible multibody dynamics formulation based on the floating frame of reference. Other formulations using super-elements allow maintaining the internal modal information [23]. In Reference [24], a dynamic spline formulation suitable for multibody dynamics implementation of flexible components was deduced.

This paper introduces a new PRBM that possesses a higher accuracy in static and quasi-static analyses compared to those reported in previous studies. This study is structured in six sections. After this introduction, the second section proposes a detailed definition of the spline PRBM. In the third section, the kinematic equations of a compliant beam are deduced through a generic approach. In the fourth, the optimization and numerical fitting methods are applied to resolve the characteristic parameters of the Spline PRBM. In the fifth, a performance comparison among the R, Revolute-Revolute (RR), PR, PRR and spline PRBM with an example in a compliant mechanism is presented. The result reveals the advantages of the novel spline PRBM in simulating flexural beams. In the last section, the conclusions are summarized.

## 2. Spline Pseudo-Rigid-Body Model

### 2.1. Dynamic Splines

**Figure 1:**
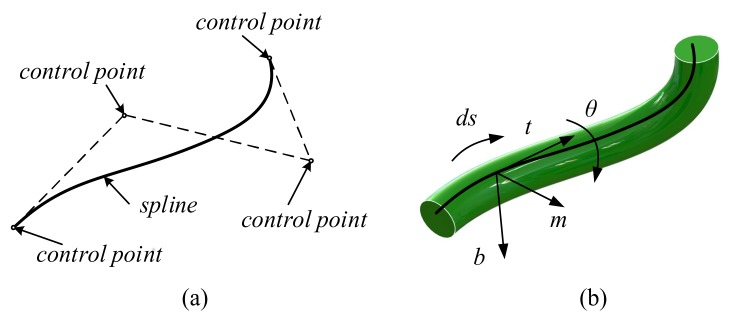
A spline model (**a**) and Frenet frame of the spline curve (**b**).

Quin [25] introduced dynamic splines in computer-aided design simulation. Dynamic splines have since been improved and specialized for multibody dynamics by Theetten [26] and Valentini [24]. The spline geometry description and physics-based constraining equations are combined based on their own research. A polynomial closed form expression is used in this study to express the displacement of a compliant beam. In general applications, the spline curve does not pass through all of the control points (shown in Figure 1a), even if its shape is influenced by the control points.

Considering the generic implementations of compliant mechanisms, a simplified modeling with only four control points can be used, thus ensuring simulation accuracy. In addition, the widely used Bezier interpolants will be applied in this study.

The parametric expression p(u) of a spline curve with four control points $\left\{P_{0}\ldots P_{3}\right\}$ can be written as:

(1) $$p(u)={\left(1-u\right)}^{3}P_{0}+3u{\left(1-u\right)}^{2}P_{1}+3u^{2}(1-u)P_{2}+u^{3}P_{3}$$

where the parametric interval is 0≤u≤1.

### 2.2. RR Pseudo-Rigid-Body Model

**Figure 2:**
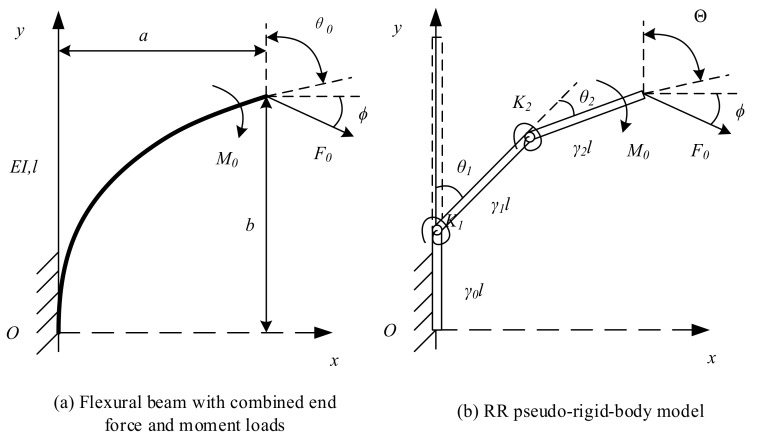
A large deformation flexural beam (**a**) and the RR PRBM (**b**).

Figure 2a shows a large deformation flexural beam subjected to combined moment loads and forces. In Figure 2b, a RR PRBM simulating the large deflection of the beam is shown. In RR PRBM, three links connecting with two revolute (R) pairs and two torsion springs replace the flexural beam.

In Figure 2a, a and b are the end point’s coordinates, $\theta_{0}$ is the deflection angle at the end point, l is the original length, and ∅ is the angle of the force direction about the *X*-axis. In Figure 2b, the length of three links in the model are $\gamma_{i}l\left(i=0,1,2\right)$, where $\gamma_{i}$ is the characteristic radius factor, satisfying $\gamma_{0}+\gamma_{1}+\gamma_{2}=1$. $K_{1}$ and $K_{2}$ are the stiffness of the torsion spring. These constants and coefficients are the main characteristic parameters of the RR PRBM.

### 2.3. Spline Pseudo-Rigid-Body Model

**Figure 3:**
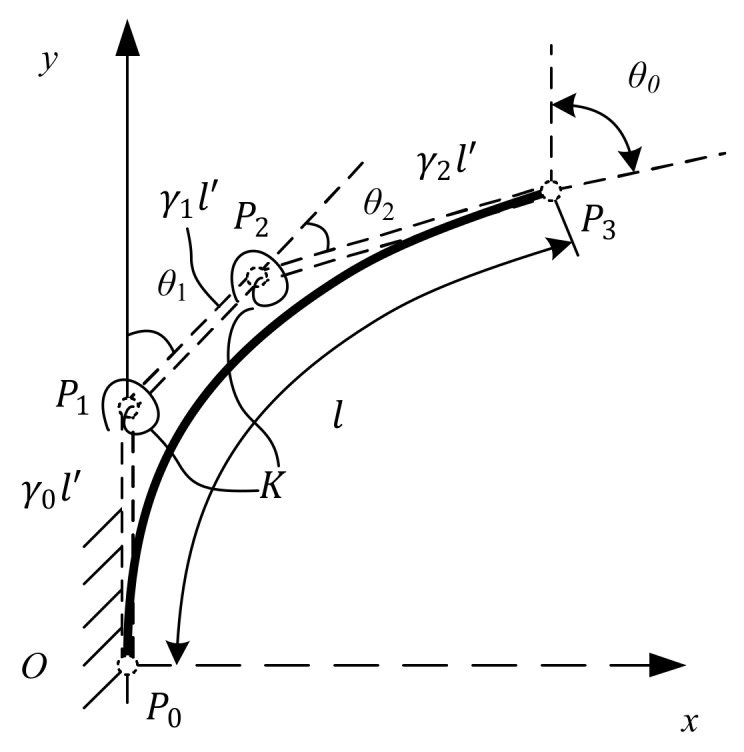
The spline pseudo-rigid-body model.

Figure 3 shows a flexural beam with combined loads at its end that corresponds to the spline PRBM. The flexural beam is replaced by a spline curve with four control points ($P_{0},\;P_{1},\;P_{2},\;P_{3}$), as well as the four control points from the three rigid links. The resistances of the flexural beam’s deflection are represented by torsion springs. Considering that the length of the spline curve after bending needs to be equal to the original length of the spline curve, then the sum of the three rigid links becomes l′. Referring to the RR PRBM, the rigid link’s length in the spline PRBM is $\gamma_{i}l\prime \left(i=0,1,2\right)$. The product $\gamma_{i}l\prime$ is the characteristic radius. Assuming that the flexural cantilever beam is homogeneous, the torsion spring stiffness constants at the two rotation joints ($K_{1}$, $K_{2}$) should be equal to K.

Assuming that the axial deflection is neglected, the arc length of the spline is equal to the original length, as represented by the following equation:

(2) $$S=\int_{spline}ds=\int_{0}^{1}\left\|\frac{dp(u)}{du}\right\|du=l$$

## 3. Equations of Motion with Dynamic Spline Model

As shown in Figure 1b, a spline curve passes in the center of every cross-section in a beam. The movements of control points will move the spline curve and change its shape. A spline curve with four control points possesses 3 × 4 DOFs, because every point has three DOFs. Considering that the beam in Figure 1b has thickness which provide an extra dimension, the rotation of the cross-section around the neural axis can be considered as the fourth DOF of the control point. Thus, a four-parameter vector is used here to express the ith control point:

(3) $$P_{i}={\left\{\begin{array}{cccc} q_{4i-3t} & q_{4i-2} & q_{4i-1} & q_{4i} \end{array}\right\}}^{T}={\left\{\begin{array}{cccc} x_{i} & y_{i} & z_{i} & \theta_{i} \end{array}\right\}}^{T}={\left\{\begin{array}{cc} r_{i} & \theta_{i} \end{array}\right\}}^{T}$$

Based on Lagrange equations, these coordinates can be used in deducing the equations of the motion of a spline curve:

(4) $$\{\begin{array}{c} \frac{d}{dt}\frac{\partial T}{\partial {\dot{q}}_{i}}-\frac{\partial T}{\partial q_{i}}=\nabla U+\psi_{q}^{T}\lambda -F_{ext},i=1,2\ldots 4m\\ \psi =\left\{0\right\} \end{array}$$

where

T is the kinetic energy;

$\dot{q_{i}}=\frac{dq_{i}}{dt}$ is the ith generalized coordinate’s time derivative;

U is the elastic energies;

$\Psi_{q}$ is the vector’s Jacobian matrix of the constraint equations Ψ;

λ is the Lagrange multipliers’ vector associated with the constraints $\Psi_{i}$;

$F_{ext}$ is the external applied loads’ vector.

Since the spline curve is continuous, the kinetic energy T can be computed as:

(5) $$T=\frac{1}{2}{\int_{spline}\left\{\dot{p}(u)\right\}}^{T}\left[M\right]\left\{\dot{p}(u)\right\}ds$$

where

$\left[M\right]=\left[\begin{array}{cc} \begin{array}{cc} \mu & 0\\ 0 & \mu \end{array} & \begin{array}{cc} 0 & 0\\ 0 & 0 \end{array}\\ \begin{array}{cc} 0 & 0\\ 0 & 0 \end{array} & \begin{array}{cc} \mu & 0\\ 0 & I \end{array} \end{array}\right]$ is the inertia matrix;

μ is the spline curve’s linear density;

I is the cross-section’s polar moment of inertia;

s is the arc length $\left(ds=\|\frac{dp\left(u\right)}{du}\|du\right)$.

The elastic energy U can be divided into three parts: stretching $U_{stretching}$, bending $U_{bending}$, and twisting $U_{twisting}$:

(6) $$U=U_{stretching}+U_{bending}+U_{twisting}$$

They can be written as integrals, respectively:

(7) $$\{\begin{array}{c} U_{stretching}=\frac{1}{2}\int_{spline}EA{\left(\varepsilon_{s}-\varepsilon_{s}^{0}\right)}^{2}ds=\frac{1}{2}\int_{0}^{1}EA{\left(\varepsilon_{s}-\varepsilon_{s}^{0}\right)}^{2}\left\|\frac{dp(u)}{du}\right\|du\\ U_{bending}=\frac{1}{2}\int_{spline}EI{\left(\varepsilon_{b}-\varepsilon_{b}^{0}\right)}^{2}ds=\frac{1}{2}\int_{0}^{1}EI{\left(\varepsilon_{b}-\varepsilon_{b}^{0}\right)}^{2}\left\|\frac{dp(u)}{du}\right\|du\\ U_{twisting}=\frac{1}{2}\int_{spline}GI{\left(\varepsilon_{t}-\varepsilon_{t}^{0}\right)}^{2}ds=\frac{1}{2}\int_{0}^{1}GI{\left(\varepsilon_{t}-\varepsilon_{t}^{0}\right)}^{2}\left\|\frac{dp(u)}{du}\right\|du \end{array}$$

where

A is the cross-section’s area;

E is the material’s Young modulus;

I is the cross-section’s momentum of inertia;

G is the cross-section’s shear modulus;

$\varepsilon_{s}^{0}$ is the original curve’s stretching strain;

$\varepsilon_{s}$ is the deformed curve’s stretching strain;

$\varepsilon_{b}^{0}$ is the original curve’s bending strain;

$\varepsilon_{b}$ is the deformed curve’s bending strain;

$\varepsilon_{t}^{0}$ is the original curve’s twisting strain;

$\varepsilon_{t}$ is the deformed curve’s twisting strain.

All of these strains can be approximated as:

(8) $$\{\begin{array}{c} e_{s}(u)=1-\left\|\frac{dp(u)}{du}\right\|\\ e_{b}=k(u)=\frac{\left\|p\prime (u)\times p\prime \prime (u)\right\|}{{\left\|p\prime (u)\right\|}^{3}}\\ e_{t}=t(u)+\frac{dq(u)}{du}=\frac{\left(p\prime (u)\times p\prime \prime (u))\times p\prime \prime \prime (u\right)}{\left\|p\prime (u)\times p'\prime \prime (u)\right\|}+\frac{dq(u)}{du} \end{array}$$

n constraint equations $\Psi_{i}$ deduced from connections at the beam’s end can be written as geometrical relationships:

(9) $$\left\{ \begin{array}{l} \psi_{1}=0\\ \ldots \\ \psi_{n}=0 \end{array}\right.$$

All of the elements used to evaluate Equation (4) are included in Equations (6) and (9).

## 4. Characteristic Parameters

### 4.1. Kinematic Equations

The end point’s slope angle in the spline PRBM is Θ, and the two torsion spring angles are $\theta_{1}$ and $\theta_{2}$, as shown in Figure 3. $F_{x}$ is the vertical force, while $F_{y}$ represents the horizontal force. Therefore, the component forces can be expressed as follows:

(10) $$\begin{array}{l} F_{x}=F_{0}\cos\phi \\ F_{y}=F_{0}\sin\phi \end{array}$$

Because the slope angle Θ of the spline PRBM shown in Figure 3 should be equal to the deflection angle $\theta_{0}$ of the flexural beam, the following equations can be obtained:

(11) $$\begin{array}{c} \frac{a}{l\prime }=\gamma_{1}\sin\theta_{1}+\gamma_{2}\sin(\theta_{1}+\theta_{2})\\ \frac{b}{l\prime }=\gamma_{0}+\gamma_{1}\sin\theta_{1}+\gamma_{2}\sin(\theta_{1}+\theta_{2})\\ \theta_{0}=\theta_{1}+\theta_{2} \end{array}$$

Therefore, the two torsion spring angles $\theta_{1}$ and $\theta_{2}$ can be derived from the equation above:

(12) $$\begin{array}{c} \theta_{1}=\sin^{-1}(\frac{(b/l\prime )-\gamma_{2}\sin\theta_{0}}{\gamma_{1}})\\ \theta_{2}=\theta_{0}-\sin^{-1}(\frac{(b/l\prime )-\gamma_{2}\sin\theta_{0}}{\gamma_{1}}) \end{array}$$

### 4.2. Static Equations

Considering the combined loads applied at the end of the spline PRBM, the lateral force and torque can be presented as follow:

(13) $$\left\{ \begin{array}{l} T_{1}=F_{x}l\prime (\gamma_{1}\cos\theta_{1}+\gamma_{2}\cos(\theta_{1}+\theta_{2}))+F_{y}l\prime (-\gamma_{1}\sin\theta_{1}-\gamma_{2}\sin(\theta_{1}+\theta_{2}))+M_{0}\\ T_{2}=F_{x}l\prime \gamma_{2}\cos(\theta_{1}+\theta_{2})-F_{y}l\prime \gamma_{2}\sin(\theta_{1}+\theta_{2})+M_{0} \end{array}\right.$$

The torques $T_{1}$, $T_{2}$ are the product of the angle $\theta_{1}$, $\theta_{2}$ and the torsion spring stiffness constant $K_{1}$, $K_{2}$, respectively:

(14) $$\left\{ \begin{array}{l} T_{1}=K\theta_{1}\\ T_{2}=K\theta_{2} \end{array}\right.$$

From Equation (14), we have:

(15) $$\left[\begin{array}{cc} \theta_{1} & 0\\ 0 & \theta_{2} \end{array}\right]\left[\begin{array}{c} K_{1}\\ K_{2} \end{array}\right]=\left[\begin{array}{ccc} \gamma_{1}\cos\theta_{1}+\gamma_{2}\cos\theta_{0} & -\gamma_{1}\sin\theta_{1}-\gamma_{2}\sin\theta_{0} & 1\\ \gamma_{2}\cos\theta_{0} & -\gamma_{2}\sin\theta_{0} & 1 \end{array}\right]\left[\begin{array}{c} F_{x}l\prime \\ F_{y}l\prime \\ M_{0} \end{array}\right]$$

Let:

(16) $$A=\left[\begin{array}{ccc} \gamma_{1}\cos\theta_{1}+\gamma_{2}\cos\theta_{0} & -\gamma_{1}\sin\theta_{1}-\gamma_{2}\sin\theta_{0} & 1\\ \gamma_{2}\cos\theta_{0} & -\gamma_{2}\sin\theta_{0} & 1 \end{array}\right]$$

And:

(17) $$\left[\begin{array}{c} K_{1}\\ K_{2} \end{array}\right]=\left[\begin{array}{cc} \frac{1}{\theta_{1}} & 0\\ 0 & \frac{1}{\theta_{2}} \end{array}\right]A\left[\begin{array}{c} F_{x}l\prime \\ F_{y}l\prime \\ M_{0} \end{array}\right]$$

The resistance to flexibility and deflection can be replaced by the dimensionless torsion spring stiffness coefficient $K_{\theta }$, as follows:

(18) $$K=\frac{EI}{l\prime }K_{\theta }$$

Therefore:

(19) $$\left[\begin{array}{cc} \frac{EI}{l\prime } & 0\\ 0 & \frac{EI}{l\prime } \end{array}\right]\left[\begin{array}{c} K_{\theta 1}\\ K_{\theta 2} \end{array}\right]=\left[\begin{array}{cc} \frac{1}{\theta_{1}} & 0\\ 0 & \frac{1}{\theta_{2}} \end{array}\right]A\left[\begin{array}{c} F_{x}l\prime \\ F_{y}l\prime \\ M_{0} \end{array}\right]$$

It can be obtained from the above equations that:

(20) $$\left[\begin{array}{c} K_{\theta 1}\\ K_{\theta 2} \end{array}\right]=\left[\begin{array}{c} \frac{F_{x}l\prime^{2}}{EI\theta_{1}}(\gamma_{1}\cos\theta_{1}+\gamma_{2}\cos\theta_{0})+\frac{F_{y}l\prime^{2}}{EI\theta_{1}}(-\gamma_{1}\sin\theta_{1}-\gamma_{2}\sin\theta_{0})+\frac{M_{0}l\prime }{EI\theta_{1}}\\ \frac{F_{x}l\prime^{2}}{EI\theta_{2}}(\gamma_{2}\cos\theta_{0})+\frac{F_{y}l\prime^{2}}{EI\theta_{2}}(-\gamma_{2}\sin\theta_{0})+\frac{M_{0}l\prime }{EI\theta_{2}} \end{array}\right]$$

However, as mentioned in Section 2.3, the stiffness of the torsion spring at the two pin joints ($K_{1}$, $K_{2}$) are the same, so:

(21) $$K_{1}=K_{2}=K,K_{\theta 1}=K_{\theta 2}=K_{\theta }$$

There are, in total, five characteristic parameters in the spline PRBM: the characteristic radius factors $\gamma_{0}$, $\gamma_{1}$, $\gamma_{2}$, the torsion spring stiffness coefficient $K_{\theta }$, and the ratio $l^{\prime }/l$. These five characteristics will be further discussed in the following section.

### 4.3. Optimal Characteristic Parameters

**Figure 4:**
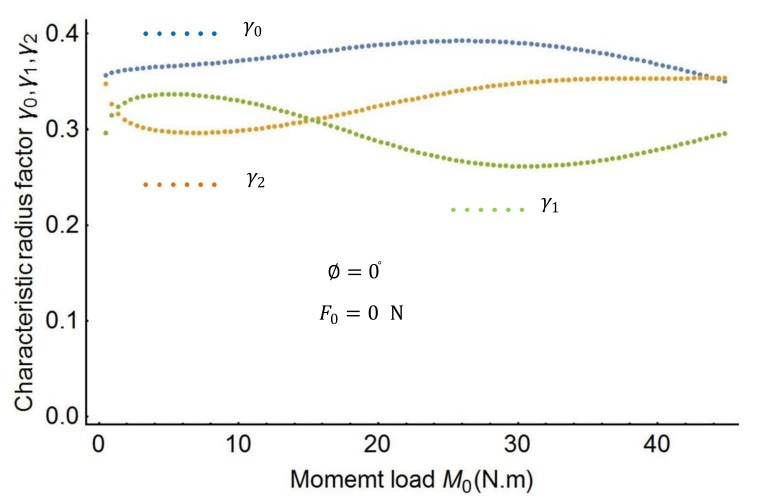
Characteristic radius factors as a function of the moment.

**Figure 5:**
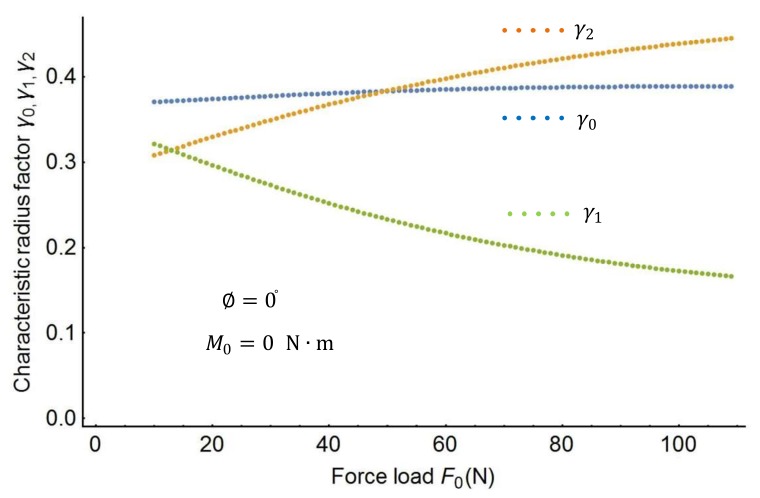
Characteristic radius factors as a function of the force.

**Figure 6:**
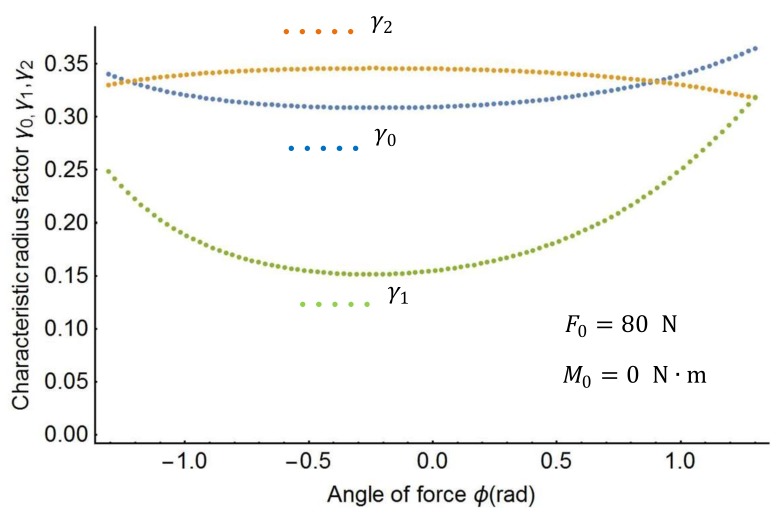
Characteristic radius factors as a function of the force angle.

In this section, this study proposes an effective approach to obtain the spline PRBM’s characteristic parameters. The effective approach uses the following steps. First, the characteristic radius factors $\gamma_{0}$, $\gamma_{1}$, $\gamma_{2}$ in the spline PRBM are investigated by varying force and moment. Figure 4 shows the plots of the characteristic factors when the moment $M_{0}$ changes from 0 to 50 N·m. Figure 5 shows the variation of the characteristic factors when the force $F_{0}$ changes from 0 to 120 N. Figure 6 shows the plots of the characteristic factors when the force angle ∅ changes from −90° to 90°. As the figures show, the characteristic factors change monotonically as the load changes from the shape in Figure 4 to that in Figure 6. It is therefore necessary to find out the characteristic factors through a three-dimensional search.

**Figure 7:**
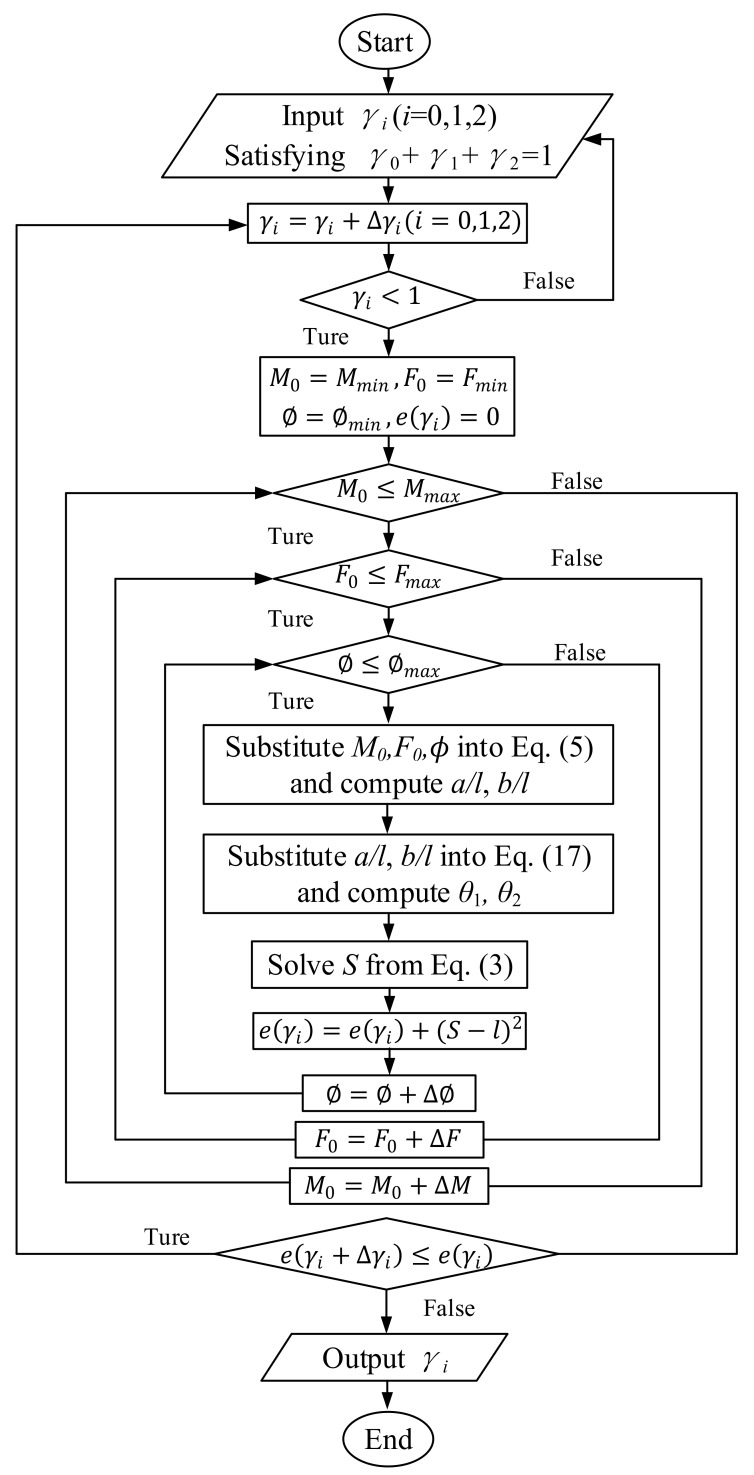
Optimization procedure chart for obtaining the spline PRBM’s characteristic parameters.

An optimization method is used here to obtain the optimal characteristic factors $\gamma_{0}$, $\gamma_{1}$, $\gamma_{2}$. The stiffness coefficient $K_{\theta }$ will be calculated later. Considering that this is a three-dimensional search, a flow chart, as shown in Figure 7, was developed to illustrate the full optimization procedure.

By using the optimization procedure, the three optimal characteristic radius factors can be determined as follows:

$$\gamma_{0}=0.36,\;\gamma_{1}=0.26,\;\gamma_{2}=0.38$$

Since the arc length of the spline should be equal to the original beam length, the effects of revolute angles $\theta_{1}$, $\theta_{2}$ on the length ratio $l^{\prime }/l$ will be investigated in second step. To do this, the present study proposes using the polynomial fitting model. The fitting model is presented in Equation (22).

(22) $$\frac{l\prime }{l}=1-0.0226\theta_{1}-0.238\theta_{2}+0.0786\theta_{1}{}^{2}+0.0813\theta_{2}{}^{2}+0.0643\theta_{1}\theta_{2}$$

**Figure 8:**
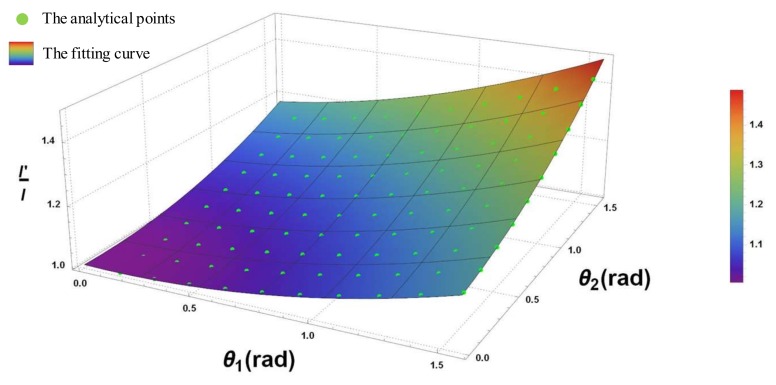
Nonlinear regression of length coefficient.

Figure 8 shows the analytical points and the fitting curve. Also from Figure 8, it can be seen that all of the analytical points fall on the fitting surface:

### 4.4. Optimal Spring Stiffness Coefficients

**Figure 9:**
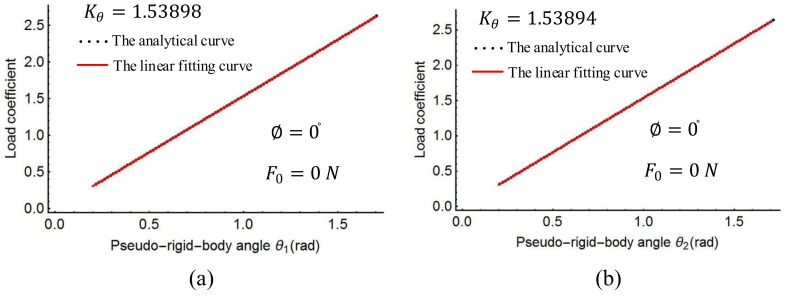
Linear regression of stiffness coefficient with action moment: (**a**) rotation joints *P*_1_, (**b**) rotation joint *P*_2_.

**Figure 10:**
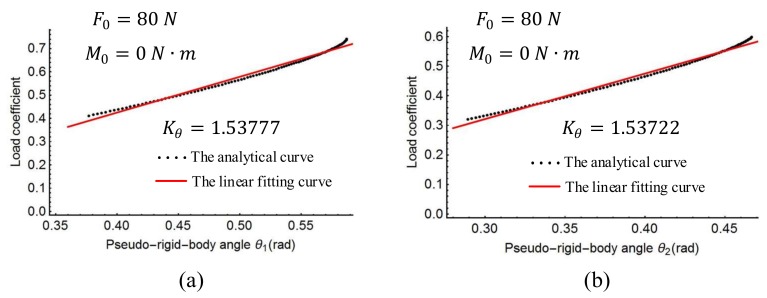
Linear regression of stiffness coefficient with action force angle: (**a**) rotation joints *P*_1_, (**b**) rotation joint *P*_2_.

**Figure 11:**
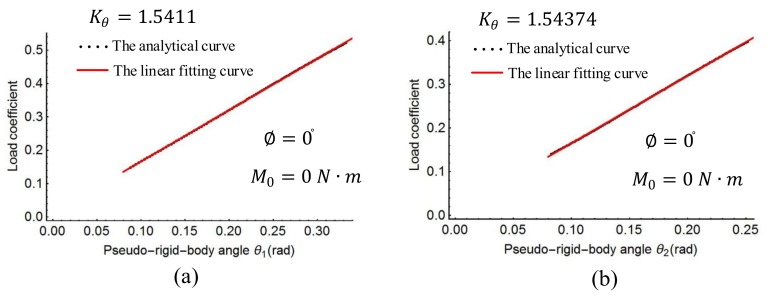
Linear regression of stiffness coefficient with action force: (**a**) rotation joints *P*_1_, (**b**) rotation joint *P*_2_.

The characteristic radius factors $\gamma_{0}$, $\gamma_{1}$, $\gamma_{2}$ and the length ratio $l^{\prime }/l$ of the spline PRBM were obtained as shown above. The torsion spring stiffness coefficient $K_{\theta }$ in Equation (20) was then determined with a linear regression process. The linear fitting curve is shown in Figure 9, Figure 10 and Figure 11. Figure 9 shows the plots of the load coefficient and rotational angles when the moment $M_{0}$ changes from 0 to 50 N·m. Figure 10 shows the load coefficient and rotational angles when the force $F_{0}$ changes from 0 to 120 N. Figure 11 shows the plots of the load coefficient and rotational angles when the force angle ∅ changes from −90° to 90°. From Figure 9, Figure 10 and Figure 11, it can be seen that the spring stiffness in each of the three cases changes slightly. Replacing the stiffness of the torsion spring with the average value of these three cases, the result is as follows:

$$K_{\theta }=1.539$$

The spline PRBM is now complete, with all five characteristic parameters determined.

## 5. An Example of Application

**Figure 12:**
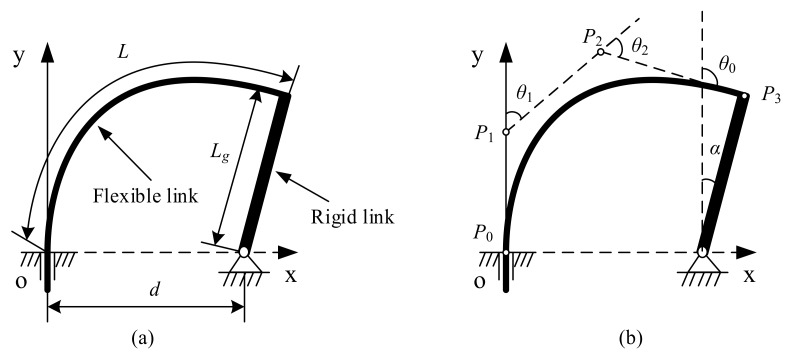
Compliant slider crank mechanism (**a**) and its spline PRBM (**b**).

In order to test the accuracy of the spline PRBM, in this section this study presents its application to a compliant mechanism. The compliant mechanism is a planar mechanism consist of a crank and a slider in which the slider is pivoted on the rigid crank and attached to the foundation frame, as shown in Figure 12a.

The effect of twisting deformation can be neglected because the mechanism is planar. Referencing Figure 12b, the following constraints must be considered:

(23) $$\begin{array}{c} P_{0,x}=0\\ P_{0,y}=0\\ P_{1,x}=0\\ \theta_{0}=\alpha +90{}^{\circ}\\ {\left(P_{3,x}-d\right)}^{2}+P_{3,y}{}^{2}-L_{g}{}^{2}=0 \end{array}$$

The dimensional variables that should be monitored are the distance d between A and O, the width b, the height h of the cross-section of the flexible beam, the length $L_{g}$ of the rigid link. The variables are given in Table 1.

**Figure 13:**
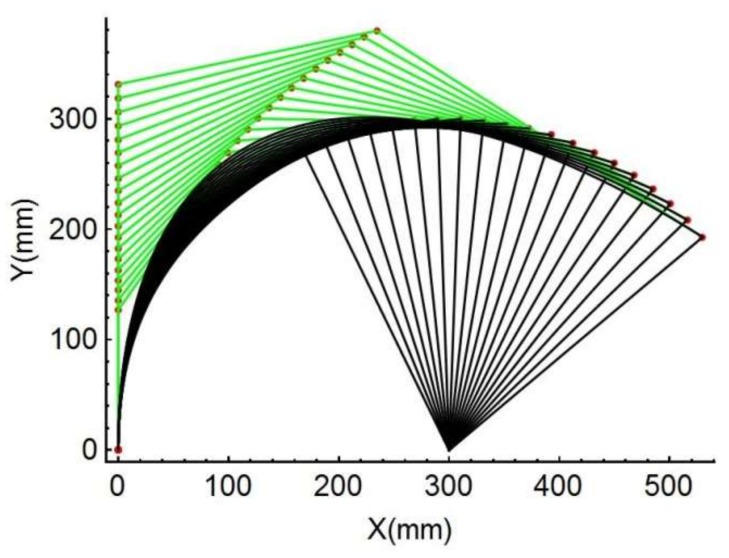
Simulation of the compliant effects during rigid beam rotation.

**Figure 14:**
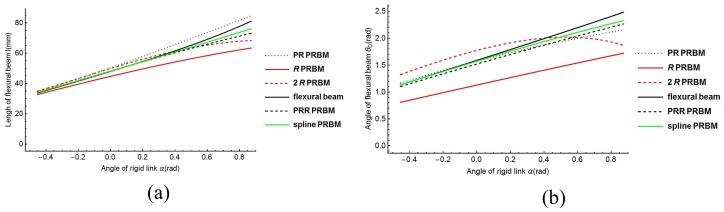

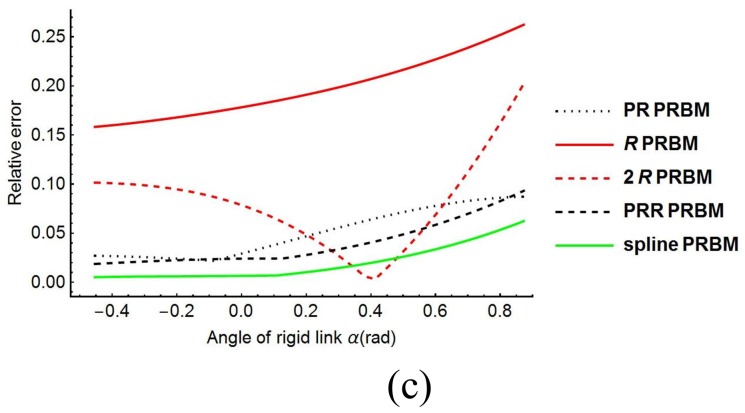
Length results (**a**), angle results (**b**), and relative errors (**c**) of PRBMs.

Once the dimensional and material variables are given, the spline PRBM can be calculated. The results can be plotted as 20 snapshots. Figure 13 shows the simulation’s visual results with the entire range of motion −30°≤α≤60°. The other PRBMs (R, RR, PR, PRR as shown in Figure 14), are calculated with the same variables shown above. Figure 14a shows the flexural beam length of the 1R, 2R, PR, PRR, and spline PRBMs. Figure 14b shows the deflection angles of the 1R, 2R, PR, PRR, and spline PRBMs. It can be seen from Figure 14a,b that the five PRBMs can closely follow the angles of the actual flexural beam in a progression of tiny deformations.

However, the results of R, RR PRBMs deviate from the actual value in the large deflection area. This is because the R, RR models possess low degrees of freedom and have low simulation accuracy. Compared with the R, RR PRBMs, the prismatic pair and compression spring improve the PRR PRBM’s accuracy. Although the PRR PRBM has great simulation accuracy, the spline PRBM’s result is much more accurate in simulation. This result indicates the effectiveness of the spline PRBM method.

This study now discusses the simulating errors of all five PRBMs to reveal further advantages of the spline PRBM. The relative angle error between the simulation’s angle and the actual angle is defined as:

(24) $$e_{r}=\frac{\left|l-l_{0}\right|}{2l_{0}}+\frac{\left|\theta -\theta_{0}\right|}{2\theta_{0}}$$

where l is the PRBMs’ flexural beam length, $l_{0}$ is the actual flexural beam’s length, θ is the PRBMs’ deflection angle, $l_{0}$ is the actual flexural beam’s deflection angle.

The relative errors of the five models are shown in Figure 14c. The maximum errors at L=20 (mm) for the R, RR, PR, PRR, and spline PRBM are 26.24%, 20.23%, 8.72%, 9.31%, and 6.23%, respectively, as listed in Table 2. As shown in Figure 14, the spline PRBM has the smallest relative error while the R and RR PRBMs have the biggest relative error, while the PR and PRR PRBMs have medium relative errors. Table 2 lists the maximum and average relative error of the five PRBMs. From Table 2, it can be seen that the tendencies of average errors and maximum errors are the same.

Based on the numerical results shown, it can be summarized that the PRR PRBM possesses a higher simulation precision in tracking the actual angle due to the high number of DOFs. When the number of DOFs is the same, a prismatic pair helps the PRR and PR PRBM to perform better than the 2R and 1R PRBM. However, the spline PRBM makes a significantly improvement in simulation.

## 6. Conclusions

This study proposed a spline PRBM based on dynamic spline and the 2R RPBM. The spline PRBM considers stretching deflection and twisting deflection as well as bending deflection, and because of its stretching, twisting, and bending deflection, the spline PRBM is also suitable for 3D structures with complex shapes. Optimization and numerical fitting methods were utilized to determine all of the spline PRBM’s characteristic parameters. This study showed the superiority of the new spline PRBM when compared with the other PRBMs. The spline PRBM provides a more accurate simulation model in static and quasi-static analyses. In addition, it can be used in dynamic simulations by employing dynamic equations. Therefore, the new spline PRBM has superior application potential in the design and analysis of force sensor systems.

## Figures and Tables

**Table 1:** The slider crank mechanism’s dimensional and material variables.

Variable	Value
$L_{g}$ the rigid link’s ideal length	300 mm
d the ideal distance between point *A* and original point *O*	300 mm
b the cross-section’s width of the flexible beam	20 mm
h the cross-section’s height of the flexible beam	2 mm
E the flexible link material’s Young modulus	1500 MPa

**Table 2:** Relative error of PRBMs.

	R (%)	RR (%)	PR (%)	PRR (%)	spline (%)
Maximum error	26.24	20.23	8.72	9.31	6.23
Average error	19.78	7.79	5.06	3.91	1.89
